# Expression of the miR-200 family in tumor tissue, plasma and urine of epithelial ovarian cancer patients in comparison to benign counterparts

**DOI:** 10.1186/s13104-020-05155-6

**Published:** 2020-07-01

**Authors:** Kalle Savolainen, Mauro Scaravilli, Antti Ilvesmäki, Synnöve Staff, Teemu Tolonen, Johanna U. Mäenpää, Tapio Visakorpi, Annika Auranen

**Affiliations:** 1 Department of Obstetrics and Gynecology, Tampere University Hospital, P.O.box 2000, 33521 Tampere, Finland; 2 Tays Cancer Centre, Tampere University Hospital and Tampere University, Tampere, Finland; 3 Institute of Biomedicine, University of Eastern Finland, Kuopio, Finland; 4 Faculty of Medicine and Health Technology, Tampere University, Tampere, Finland; 5 Fimlab Laboratories, Tampere University Hospital, Tampere, Finland

**Keywords:** Ovarian cancer, HGSOC, microRNA, Liquid biopsy, Biomarker

## Abstract

**Objective:**

Plasma, but also urine sample could represent a simple liquid biopsy for ovarian cancer biomarker detection. The miRNA-200 family has been shown to be dysregulated in ovarian cancer. The aim of this study was to isolate three members of miR-200 family from tumor tissue, plasma and urine of high-grade serous ovarian cancer patients in comparison with samples from patients with benign ovarian tumors. This is a methodological pilot study of a prospective ovarian cancer patient cohort investigating the potential of liquid biopsies and the role of miRNAs in ovarian cancer treatment.

**Results:**

MiR-200a, miR-200b and miR-200c were isolated from samples of nine ovarian cancer patients and seven patients with benign ovarian tumor. The most significant finding is that all three miRNAs were detectable in all sample types. Tumor tissue and plasma, but not urine analysis was able to discriminate malignant and benign samples. A correlation between the miRNA-200 expression in urine and plasma was observed in malignant samples only. Plasma and urine with respect to miRNA detection show potential according to this study, but larger studies are needed to clarify the usefulness of these liquid biopsies in ovarian cancer.

Trial registration: ClinicalTrials.gov NCT02758652, May 2, 2016.

## Introduction

This study is a pilot part of the ongoing registered prospective CHEMOVA study, which aims at analyzing the role of miRNAs in prediction of primary treatment response and in the development of chemoresistance in high-grade serous ovarian cancer (HGSOC). In this study, the feasibility of liquid biopsies in the diagnosis and treatment of HGSOC is examined. Being usually asymptomatic in early stages (FIGO stages I–II), the disease is usually diagnosed in advanced stages (IIIC–IV). The 5-year survival rate exceeds 90% in early stages, but in advanced stages the 5-year survival remains at a dismal 30% [1].

MicroRNAs (miRNAs) are highly conserved small non-coding RNAs that regulate gene expression with post-transcriptional silencing of the target genes [2–4]. Aberrant miRNA expression has been observed in various types of human cancers including ovarian cancer [5]. Taking into account the important regulatory roles that miRNAs have in cancer development, by acting either as oncogenes or as tumor-suppressor genes, they represent potential biomarkers in ovarian cancer [5].

The potential of urinary miRNAs in cancer diagnostics, particularly in cancers of the urogenital tract has been shown [6–8]. Weber et al. found differences in miRNA expression profiles of different human body fluids within an individual. The highest number of unique miRNA species were found in plasma, contrasting no unique miRNA species in urine [9]. Thus, it is likely that miRNAs found in urine are also found in plasma. However, no direct correlation between miRNA expression levels in blood and urine has been yet clearly demonstrated.

Hundreds of dysregulated miRNAs have been found in studies comparing the expression profiles of miRNAs in malignant ovarian tumors and normal ovaries. A number of them have been shown to be dysregulated in multiple independent studies [10], including members of the miR-200 family, which have been shown to be upregulated in ovarian cancer cells [11–13]. In this methodological study, we wanted to examine whether urinary miRNA expression correlates with plasma miRNA expression. For the target miRNAs, we chose miR-200a, miR-200b and miR-200c.

## Main text

### Materials and methods

This study includes 16 patients diagnosed and treated at the Department of Obstetrics and Gynecology, Tampere University Hospital, Finland between 2016 and 2018 (Table 1). The main interest was to optimize the laboratory procedures and to test the hypothesis that urine and plasma concentrations reflect each other. Thus, we selected from the CHEMOVA cohort nine HGSOC patients with large tumor mass as malignant group and seven patients with benign ovarian tumor as benign group. Patient age and BMI were similar between the groups (Student’s t-test).

**Table 1:** Patients’ data

ID	Tumor	Age^a^	BMI	Ca12–5	HE4	Stage
26	HGSOC	50′s–60′s	18.7	979	999	IIIA1 (i)
09	HGSOC	40′s–50′s	22.3	541	485	IIIB
34	HGSOC	50′s–60′s	25.0	1616	196	IIIB
28	HGSOC	50′s–60′s	33.0	1389	388	IIIC
30	HGSOC	50′s–60′s	25.4	867	979	IVB
46	HGSOC	50′s–60′s	32.6	807	220	IVB
65	HGSOC	60′s–70′s	36.4	1551	832	IVB
23	HGSOC	70′s–80′s	27.3	416	1500	≥ IIIC^b^
39	HGSOC	70′s–80′s	18.1	680	766	IVB
		md 58 av 59	md 25.4 av 27.0	md 867 av 983	md 766 av 707	
55	Cystadenoma	50′s–60′s	24.7	76	53	
17	Cystadenoma	40′s–50′s	23.9	24	164	
61	Cystadenoma	40′s–50′s	26.6	35	34	
32	Tecoma	70′s–80′s	25.1	173	NA	
35	Fibroma	50′s–60′s	37.2	69	NA	
63	Fibroma	80′s–90′s	26.6	126	56	
36	Teratoma	40′s–50′s	NA	152	46	
		md 53 av 56	md 25.8 av 27.3	md 65 av 70	md 53 av 68	

The data of the patients gathered at the stage of diagnosis and after the surgery

^a^The age of the participants is presented as a range in order to protect their anonymity, BMI, body mass index, Ca12-5, plasma Ca12-5 antigen value; HE4, plasma human epididymal antigen 4 value; Stage, stage of the ovarian cancer according to FIGO 2016 guidelines, HGSOC, high-grade serous ovarian cancer; ^b^ Inoperable patient, no thorough staging operation. md, median; av, average; NA, Not available

Tumor tissue samples were collected at the operating-room into tubes containing Tissue Tek^®^, snap frozen in liquid nitrogen and stored at − 80 °C. 10 × 10 μm sections of the samples were cut using a Leica CM3050S cryostat (Leica Microsystems GmbH, Wetzlar, Germany) and RNA was collected with TRI Reagent^®^ (Molecular Research Center Inc. Cincinnati, OH, USA) according to the manufacturer’s protocol. Moreover, 6 μm tissue sections were cut, hematoxylin & eosin stained and the percentage of area occupied by the tumor cells was evaluated by experienced gynecologic pathologist. Specimens with tumor content of > 70% or 50–70% were chosen to represent acceptable miRNA content and were included. Histological typing was conducted according to the WHO criteria.

Urine and plasma samples were collected before surgery. Urine was centrifuged at 2000 G for 15 min and plasma in EDTA tubes at 2000 G for 10 min and the supernatant was collected and stored at − 80 °C.

From duplicate 5 mL urine samples the exosomal fraction was precipitated using the Exosome Precipitation Solution (Macherey–Nagel GmbH, Düren, Germany) according to manufacturer’s instructions and the precipitated exosomes were re-suspended in 300 μL of nuclease-free water. The samples were further treated identically as described below for plasma samples.

Duplicate 300 μL plasma samples were incubated shortly with Proteinase K (30 µg/µL, 10 min, 37º C). The RNA was collected using the NucleoSpin^®^ miRNA Plasma kit (Macherey–Nagel GmbH, Düren, Germany) according to the manufacturer’s protocol. 25 fmoles of spike-in RNA (cel-miRNA-39) were added to the samples before RNA collection.

Four ng of RNA collected from plasma and urine and 25 ng of RNA collected from the tissue samples were reverse-transcribed using TaqMan^®^ MicroRNA Assay reverse-transcription probes for miRNA-200a (Assay-ID00502), miRNA-200b (Assay-ID002251), miRNA-200c (Assay-IDmiRNA-200c), cel-miRNA-39 (Assay-ID000200) and RNU6B (Assay-ID002300) and the microRNA reverse-transcription kit (Thermo Fischer Scientific, Waltham, MA, USA). The qRT-PCR was performed using TaqMan^®^ Assay probes (assay ID listed above) and TaqMan^®^ Universal Master Mix on a BioRad CFX96 ™ Real-Time PCR equipment (Bio-Rad Laboratories, Hercules, CA). The raw expression values were normalized against the spike-in RNA for plasma and urine and against RNU6B for the tissue.

## Results

The RNA extraction from the plasma samples showed a relatively high efficiency compared to the urine samples. 300 μL of plasma were used for RNA extraction. The total yields were 500–3000 ng and the final RNA concentrations were 15–100 ng/μL, depending on sample. Five mL of urine were used from each sample for the exosome precipitation and subsequent RNA extraction from the exosomal fraction. In this case the total yield was 60–300 ng of exosomal RNA, with concentrations of 2–9 ng/µL. Nonetheless, the RNA collected from the exosomal fraction of the urine was sufficient in most cases for the detection of the target miRNAs at qRT-PCR level.

From 16 tissue samples obtained from nine HGSOC patients, eight showed tumor cell percentage of > 70. Five samples with a percentage of 50–70 were also included. Three samples were excluded. Total of 15 benign ovarian tumor samples from seven patients were included. The RNA extraction from these samples showed an average concentration of 400 ng/µL, and an average total yield of 8000 ng of RNA.

**Fig. 1:**
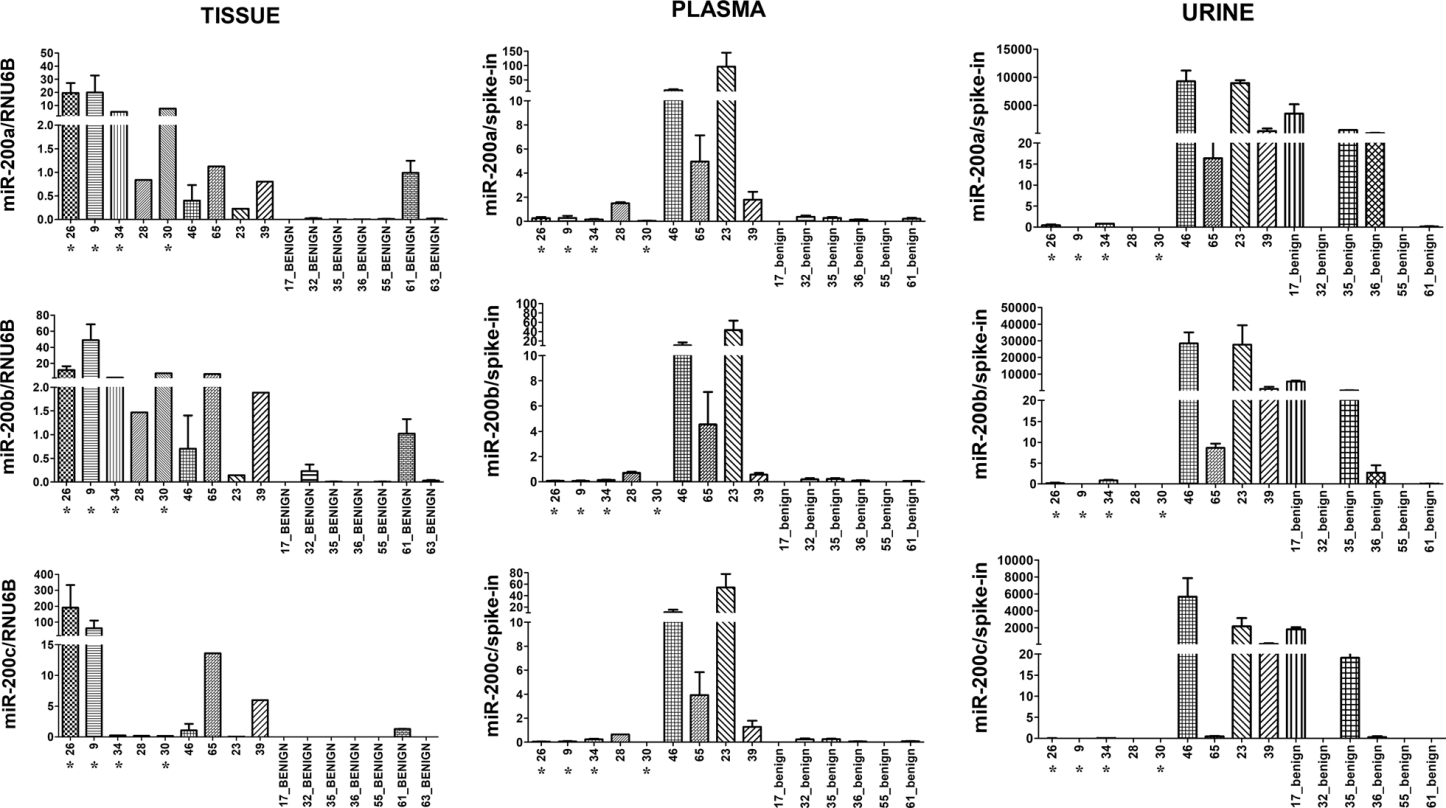
Expression of miR-200a, miR-200b and miR-200c in clinical samples. Relative expression levels of miR-200a, miR-200b and miR-200c in plasma, urine and tumor tissue of the HGSOC patients and patients with benign ovarian tumors (the graphs show mean and SD). HGSOC patients marked with asterisk (*) had no extensive peritoneal carcinosis in addition to ovarian tumors as did rest of the cancer patients. Except patient 30 they had lower stage of the disease (IIIA or IIIB) compared to rest of the HGSOC patients (IIIC or IVB). Patient 30 had liver metastasis of 6 cm, which raised the stage into IVB. The expression levels in tissue samples are normalized against RNU6B. The expression levels in plasma and urine samples are normalized against spiked-in cel-miR-39. The liquid samples from patient 63 were not available

The expression analysis performed on tissue, plasma and urine samples shows that miR-200a, miR-200b and miR-200c were consistently expressed and had very similar profiles within each sample type, suggesting co-expression in both malignant and benign cases (Fig. 1). The detection of all three miRNAs was successful in all HGSOC plasma samples, although three out of nine patients (9, 26 and 30) had very low levels. They had no extensive peritoneal carcinosis. In the urine, however, one out of nine HGSOC patients (9) had non-detectable levels of these miRNAs. None of the patients had compromised renal function. All three miRNAs were detected in all tissue samples.

The benign samples showed very low detected levels of miR-200a, miR-200b and miR-200c in tissue and plasma samples compared to the malignant cases. In urine samples, however, two patients (17 and 35) showed significantly higher levels than the other samples, therefore showing more variation and inconsistent patterns of expression in this type of samples.

**Fig. 2:**
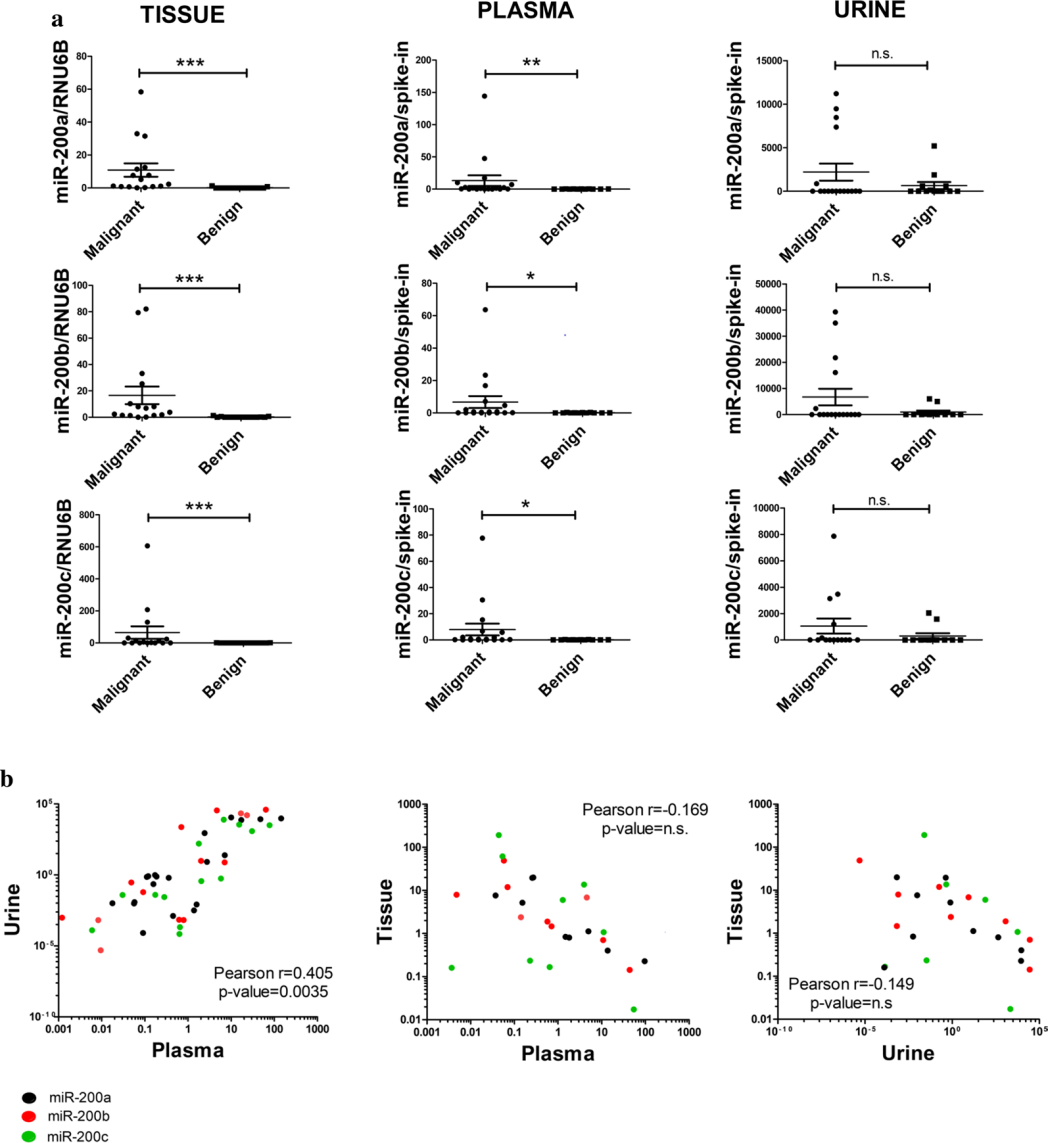
**a** Relative levels of miR-200a, miR-200b and miR-200c between malignant and benign samples. **b** Correlation of expression levels between plasma, urine and tumor tissue samples. **a** Relative expression of miR-200a, miR-200b and miR-200c in plasma and tissue are significantly higher in malignant samples compared to benign. The difference is not significant in urine samples (** p < 0.001, *** p < 0.0001). **b** Combined scatter plots showing correlations of relative expression for all three miRNAs (miR-200a (black dots), miR-200b (red dots) and miR-200c (green dots)) between plasma, urine and tumor tissue samples of HGSOC patients (Pearson’s rank correlation coefficient). For plasma and urine two replicates per sample were available, but for tissue variable numbers of replicates were available. Thus, the correlation plot for urine vs plasma was done replicate by replicate, but the correlation plots Tissue vs Plasma and Tissue vs Urine were done on average values one-to-one, explaining fewer dots in these plots. Pearson’s ranks for small sample size of individual miR-200a, miR-200b, or miR-200c did not reach significance (data not shown)

The relative expression levels of the miRNAs were significantly higher in malignant tissue samples, compared to benign (P < 0.001). Similarly, significantly higher expression was found in malignant plasma samples compared to benign counterparts (Fig. 2a). However, no significant difference was found between the expression levels of malignant and benign urine samples.

Scatter plots of correlation in the miR-200 levels between the respective plasma, urine or tissue samples are shown in Fig. 2b. We found a correlation in relative expression profile of all three miRNAs between plasma and urine samples in the patients with a malignant tumor (Pearson r = 0.405, p = 0.0035). The expression levels of the miRNAs in tissue samples did not correlate with the levels in liquid biopsies in neither group. Moreover, in the patients with benign tumors, the expression levels in plasma and urine did not correlate with each other (data not shown).

## Discussion

The expression of three members of the miR-200 family was analyzed in plasma, urine and tumor tissue collected from HGSOC patients and patients with benign ovarian tumor. The aim was to assess possible similarities in the expression profile of these commonly de-regulated miRNAs between the different sample types and, whether urine samples could replace or complement blood samples in miRNA biomarker analysis.

MiR-200a, mir-200b and miR-200c levels showed similar trend in all patients based on sample type, suggesting that they are co-expressed at similar levels. The relative levels of the miRNAs in urine and plasma were found to be comparable in the samples obtained from the patients with a malignant tumor, with most patients showing high miRNA expression in plasma also having high miRNA expression in urine. Tissue and plasma miR-200 analysis could distinguish malignant and benign cases. Moreover, this pilot study shows that plasma and urine miR-200 expression levels correlate in HGSOC but not in benign cases. This suggests that the role of urine samples, at least in parallel with plasma samples, should be further investigated in miRNA biomarker detection in HGSOC.

Small-nuclear RNAs (snRNAs) are commonly used as reference genes for miRNA expression normalization. Thus, tumor tissue expression values were normalized against RNU6B. However, several studies have reported differential expression of snRNAs in cancer and the suitability of each snRNA should be assessed for each individual experimental setup [14, 15]. The expression data of plasma and urine samples were normalized against an exogenous spike-in reference miRNA (cel-miR-39) since there is currently no consensus on appropriate endogenous reference to be used for these sample types. The miRNA expression in urine samples showed the overall largest range of variation, with most samples having relatively low expression levels, while samples from three patients with a malignant tumor and two of the controls showing very high relative levels. In future, better normalization strategies and/or absolute quantification PCR can improve the reliability of the results for liquid biopsies.

The relative expression levels in tumor tissues were not comparable with the liquid biopsies. In several cases, however, an opposite expression trend, was observed with patients having high miR-200 levels in both plasma and urine, showing low expression in tissue samples. This could be interpreted as an active secretion of miRNAs from tumor tissue, but such straightforward conclusion cannot be made from the present data taking into account that most of the patients having low expression in liquids had no extensive peritoneal carcinosis in addition to tumors. Exosomes are lipoprotein complexes acting as small membranous vesicles. The factors defining the fate of given miRNA to be secreted in exosomes are still largely unknown. Epithelial ovarian cancer (EOC) neoplastic cells have been shown to have an enhanced exosomal output as compared to normal epithelial cells [16]. Based on in vitro findings with chemoresistant ovarian carcinoma cells, it has been postulated that the release of exosomes may be a mechanism by which neoplastic EOC cells could ‘educate’ each other, thereby enhancing the development of platinum-resistant disease [17]. It will be interesting to test this hypothesis of possible predictive value of high miRNA secretion in liquid biopsies versus tumor tissue in the larger CHEMOVA cohort. We isolated total miRNA from plasma, but from urine the exosomal fraction was used. The high levels of RNase in urinary tract leads to degradation of free miRNAs and practically only exosomal miRNAs remain detectable in urine [18–20].

In conclusion, this study aimed to assess the feasibility of liquid biopsies in miRNA expression profiling in HGSOC. The most significant finding is that the three members of the miR-200 family are detectable in urine, plasma and tissue samples obtained from the same ovarian cancer patients. Tumor tissue and plasma analysis could discriminate malignant and benign ovarian samples. Moreover, a correlation was observed between miR-200 expression in urine and plasma of ovarian cancer patients, but not in patients with benign tumor. This pilot study showed that plasma and urine as liquid biopsies could be useful in miRNA biomarker analyses in HGSOC, but more studies are needed to validate the current findings in larger cohorts of patients, including patients with early stage disease.

## Limitations

The low number of patient samples represents a limitation in interpreting the statistical findings and evaluating the methods used. RNU6B should be further evaluated as a suitable reference for normalization of qRT-PCR results in HGSOC tumor samples. Also, the higher variation in relative miRNA expression levels in urine samples could be due to a lack of effective methods for normalization of the initial exosome content and precipitation efficiency.

## Data Availability

The datasets supporting the conclusions of this article are included within this article and images. Raw data are available from the corresponding author on reasonable request.

## Abbreviations

HGSOC: High-grade serous ovarian cancer
EOC: Epithelial ovarian cancer
FIGO: International Federation of Gynecology and Obstetrics
miRNA, miR: microRNA
WHO: World Health Organisation
snRNA: Small-nuclear RNA
qRT-PCR: Quantitative reverse-transcription polymerase chain reaction
